# Van der Waals Epitaxy of Bismuth‐Based Multiferroic Layered Supercell Oxide Thin Films Integrated on Flexible Mica Substrate

**DOI:** 10.1002/smsc.202300244

**Published:** 2023-12-28

**Authors:** Jianan Shen, Benson Kunhung Tsai, Yizhi Zhang, Ke Xu, James P. Barnard, Zedong Hu, Xinghang Zhang, Haiyan Wang

**Affiliations:** ^1^ School of Materials Engineering Purdue University West Lafayette IN 47907 USA; ^2^ Elmore Family School of Electrical and Computer Engineering Purdue University West Lafayette IN 47907 USA

**Keywords:** Bi_2_NiMnO_6_ films, flexible electronics, layered supercell structures, mica, multiferroics

## Abstract

Bi_2_NiMnO_6_ (BNMO) epitaxial thin films with a layered supercell (LSC) structure have emerged as a promising single‐phase multiferroic material recently. Because of the required strain state for the formation of the LSC structures, most of the previous BNMO films are demonstrated on rigid oxide substrates such as SrTiO_3_ and LaAlO_3_. Here, the potential of BNMO films grown on muscovite mica substrates via van der Waals epitaxy, spotlighting their suitability for cutting‐edge flexible device applications is delved. Comprehensive scanning transmission electron microscopy/energy‐dispersive X‐ray analyses reveal a layered structure in the BNMO film and a pristine interface with the mica substrate, indicating high‐quality deposition and minimal interfacial defects. Capitalizing on its unique property of easily cleavable layers due to weak van der Waals forces in mica substrates, flexible BNMO/mica samples are fixed. A standout feature of the BNMO film grown on mica substrate is its consistent multiferroic properties across varied mechanical conditions. A novel technique is introduced for thinning the mica substrate and subsequent transfer of the sample, with post‐transfer analyses validating the preserved structural and magnetic attributes of the film. Overall, this study illuminates the resilient multiferroic properties of BNMO films on mica, offering promising avenues for their integration for next‐generation flexible electronics.

## Graphical abstract

This study explores the epitaxial growth of BNMO films on mica substrates through van der Waals epitaxy, highlighting their potential in flexible electronics. It emphasizes the consistent multiferroic properties of the films, notably under varied mechanical conditions, and introduces an innovative method for mica substrate thinning, paving the way for advanced flexible device applications.

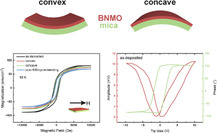

## 1 Introduction

Multiferroics have captured significant scientific interests due to their unique ability to simultaneously exhibit dual ferroic orderings, notably the cohabitation of ferroelectric and ferro‐/ferrimagnetic properties.^[^ ^1^, ^2^, ^3^, ^4^ ^]^ However, the inherent contradiction in d‐orbital occupancy between ferroelectric and magnetic materials presents a formidable challenge in realizing single‐phase multiferroic materials.^[^ ^5^ ^]^ A key quest in this field is the identification of materials that display robust magnetization and polarization at room temperature. In this context, bismuth‐based transition metal oxides have emerged as promising candidates.^[^ ^6^, ^7^, ^8^, ^9^, ^10^, ^11^, ^12^ ^]^ For instance, BiFeO_3_ in its thin‐film form, with the space group *P4mm*, was recognized as the inaugural room‐temperature multiferroic single‐phase material. While BiFeO_3_ has gained considerable attention, its intrinsic shortcomings, predominantly its antiferromagnetic character with modest magnetization, are undeniable.^[^ ^13^, ^14^, ^15^ ^]^ Another versatile single‐phase multiferroic material group is the layered supercell (LSC) oxides, including Bi_3_Fe_2_Mn_2_O_*x*_ (BFMO322), Bi_2_NiMnO_6_ (BNMO), Bi_2_AlMnO_6_ (BAMO), and others.^[^ ^11^, ^16^, ^17^ ^]^ For example, BNMO has been processed into bulk, nanoparticles, and thin‐film forms, and has been demonstrated as a promising candidate in the multiferroic community.^[^ ^18^, ^19^, ^20^, ^21^, ^22^ ^]^ Building on these prior works, in 2019, Li et al. grew BNMO epitaxial thin films on SrTiO_3_ (STO) and LaAlO_3_ (LAO) substrates, which exhibited a tunable LSC microstructure and consistent room‐temperature magnetism.^[^ ^16^ ^]^ In another work, Shen et al. innovatively fabricated freestanding BNMO thin films using a buffer stack of a CeO_2_ layer and a sacrificial Sr_3_Al_2_O_6_ layer, subsequently transferring the film onto a Si wafer while retaining the LSC architecture.^[^ ^23^ ^]^ Both the pristine and transferred BNMO films exhibited room‐temperature multiferroicity, underscoring the potential of integrating BNMO films with Si‐based electronics. Hence, LSC BNMO films present exciting prospects for probing multiferroic phenomena and enabling practical technological applications.

The development of flexible, wearable, and portable electronic devices has garnered significant scientific attention due to their transformative potential in daily life, with applications spanning health monitoring, flexible displays, and Internet of Things (IoT) devices.^[^ ^24^, ^25^, ^26^, ^27^, ^28^ ^]^ Epitaxial thin films of functional complex oxides exhibit diverse and captivating properties, stemming from the interplay of spin, charge, lattice, and orbital degrees of freedom.^[^ ^29^, ^30^, ^31^ ^]^ Consequently, these films have become focal points in electronics, photonics, and spintronics research.^[^ ^32^, ^33^, ^34^, ^35^ ^]^ Given this context, integrating functional oxides in flexible device designs is intriguing and crucial. Currently, most oxide thin films have been grown on rigid single‐crystalline oxide substrates such as STO and MgO, which are not suitable for the flexible device applications.^[^ ^16^, ^36^, ^37^ ^]^ Therefore, identifying a viable method to incorporate these remarkable oxide materials into flexible device designs remains a formidable task for the research community. To address this, two promising strategies have emerged, that is, “freestanding thin films” and “flexible substrates”. The former entails depositing films on a sacrificial buffer layer, followed by its removal and the transfer of the film to a flexible substrate.^[^ ^38^, ^39^ ^]^ Some of the challenges include chemical residues during the transfer process that could impair film performance. The latter approach typically employs a prepolished flexible metal foil as the substrate. However, large lattice mismatch and interdiffusion issues at the film–substrate interface are both major issues.^[^ ^40^, ^41^ ^]^ Thus, there is a demand for substrates that can withstand high temperatures and remain chemically inert. Muscovite mica, an inert phyllosilicate mineral composed of aluminum and potassium with the chemical formula (KF)_2_(Al_2_O_3_)_3_(SiO_2_)_6_(H_2_O) and a melting point of 1300 °C, possesses a distinct layered structure held together by weak van der Waals (vdW) forces. This weak interaction allows mica to be easily split into ultrathin layers, rendering its flexibility.^[^ ^42^ ^]^ Several oxide thin films have been effectively deposited on mica using the vdW epitaxy mechanism.^[^ ^43^, ^44^, ^45^, ^46^ ^]^ Due to its flexibility and remarkable thermal and chemical resilience, mica stands out as an ideal substrate for direct oxide thin film deposition.

**Figure 1:**
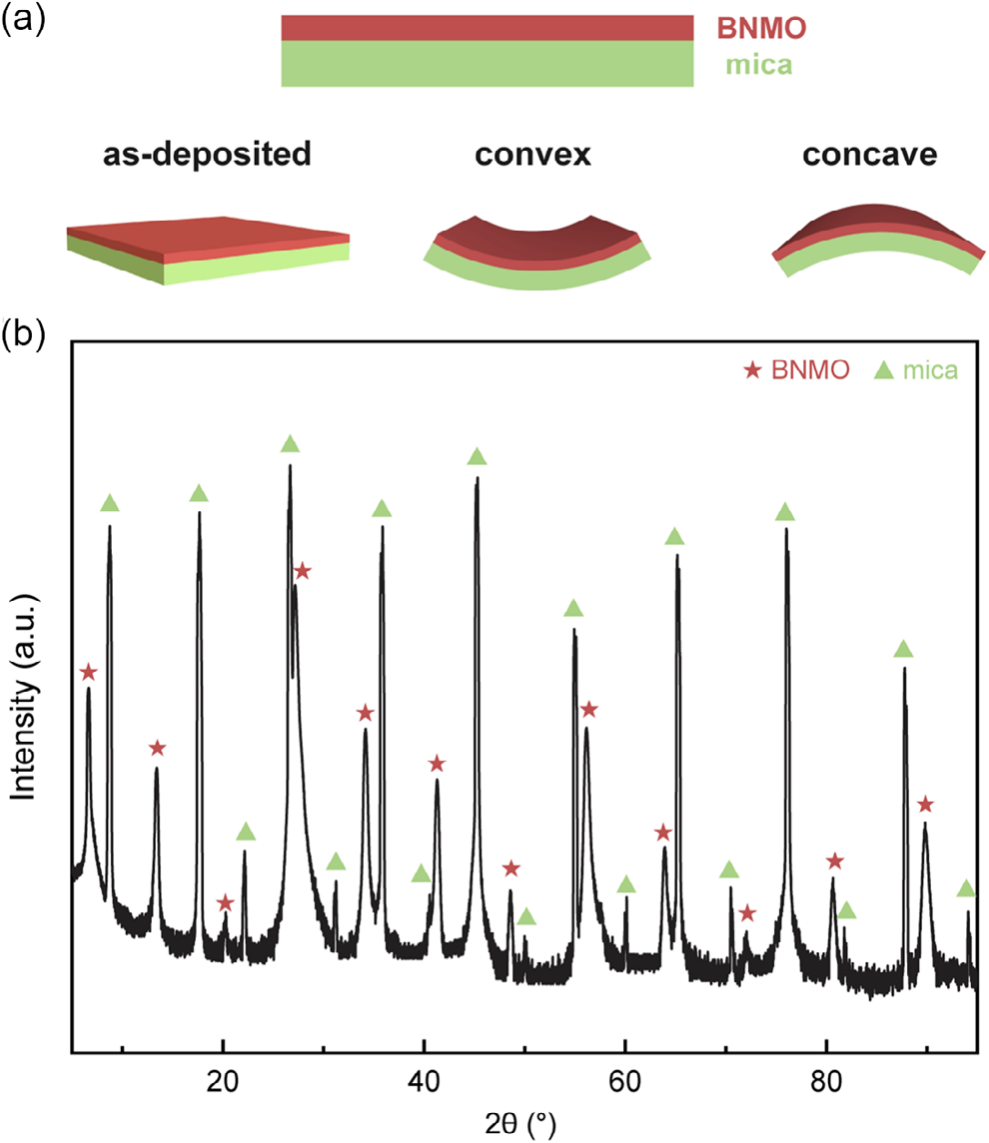
a) Schematic drawings show the film architecture and samples under different conditions. b) XRD 2*θ*‐*ω* scan result of the BNMO/mica sample.

In this work, we aim to grow BNMO epitaxial thin films with an LSC structure on a mica substrate via the vdW epitaxy. The crystallinity and microstructure of the film are meticulously characterized using X‐ray diffraction (XRD) and high‐resolution scanning transmission electron microscopy (HRSTEM). Notably, the flexibility of mica improves as its thickness reduces; thus, the mica substrate is cleaved to a thickness that displays excellent flexibility (≈50 μm). To evaluate the sample's suitability for flexible device applications, its properties are examined under four distinct mechanical conditions: as‐deposited (i.e., no bending), convex, concave, and post‐500‐cycle bending, as illustrated in schematic drawings in **Figure** **1**a. The magnetic properties, particularly saturation magnetization and coercive field, are thoroughly assessed across these conditions, followed by a comprehensive analysis of the findings. Additionally, the ferroelectric properties are systematically compared. For the material to be viable in flexible device applications, it's imperative that these properties remain relatively consistent across varying bending conditions. Finally, we further refine the method to thin the mica substrate using a water‐soluble tape while ensuring the film's structure and properties remain intact. A standout feature of this method is the ability to control the thickness of the mica substrate. By exploring the integration of BNMO LSC film on mica substrates with controlled thickness, the work paves a way for practical applications of multiferroic layered oxides in flexible spintronics.

## 2 Results and Discussion

As a layered oxide, the interlayers of mica are weakly bonded by vdW force, allowing it to be easily cleaved into thin layers. Notably, as mica is thinned down, its flexibility is significantly improved.^[^ ^44^ ^]^ In this study, we explored the potential of BNMO films for flexible device applications by investigating the properties of the BNMO film grown on mica under four different conditions: as‐deposited (no bending), convex, concave, and post‐500‐cycle bending. The as‐deposited samples were optimized for growth conditions, displaying excellent quality, supported by the XRD 2*θ*–*ω* results, as shown in Figure 1b. The XRD scan of the as‐deposited sample exhibits a highly similar pattern to a previous study of BNMO on STO substrate, that is, clear characteristic (00 L) peaks of the BNMO film spanning from 5° to 95° appearing periodically alongside the substrate peaks, indicating a comparable layered structure.^[^ ^23^ ^]^ The bandgap of the BNMO LSC film, derived from the Tauc's plot, is $E_{g}=2.78\text{ eV},$ as illustrated in Figure S2, Supporting Information, aligns well with findings from prior research.^[^ ^16^ ^]^ This intriguing phenomenon is noteworthy since LSC films typically require a large lattice mismatch and an initial strained state to transition from the pseudocubic to the LSC form.^[^ ^47^ ^]^ However, in this case, the LSC film can grow smoothly on mica in the absence of strong chemical bonding via vdW epitaxy mechanism. A schematic drawing of the crystal model showing the structural relationship between the BNMO film and the mica substrate is included in Figure S1, Supporting Information, influenced by the previous study.^[^ ^48^ ^]^ A comprehensive investigation and discussion of the film's microstructure are presented in subsequent sections.

**Figure 2:**
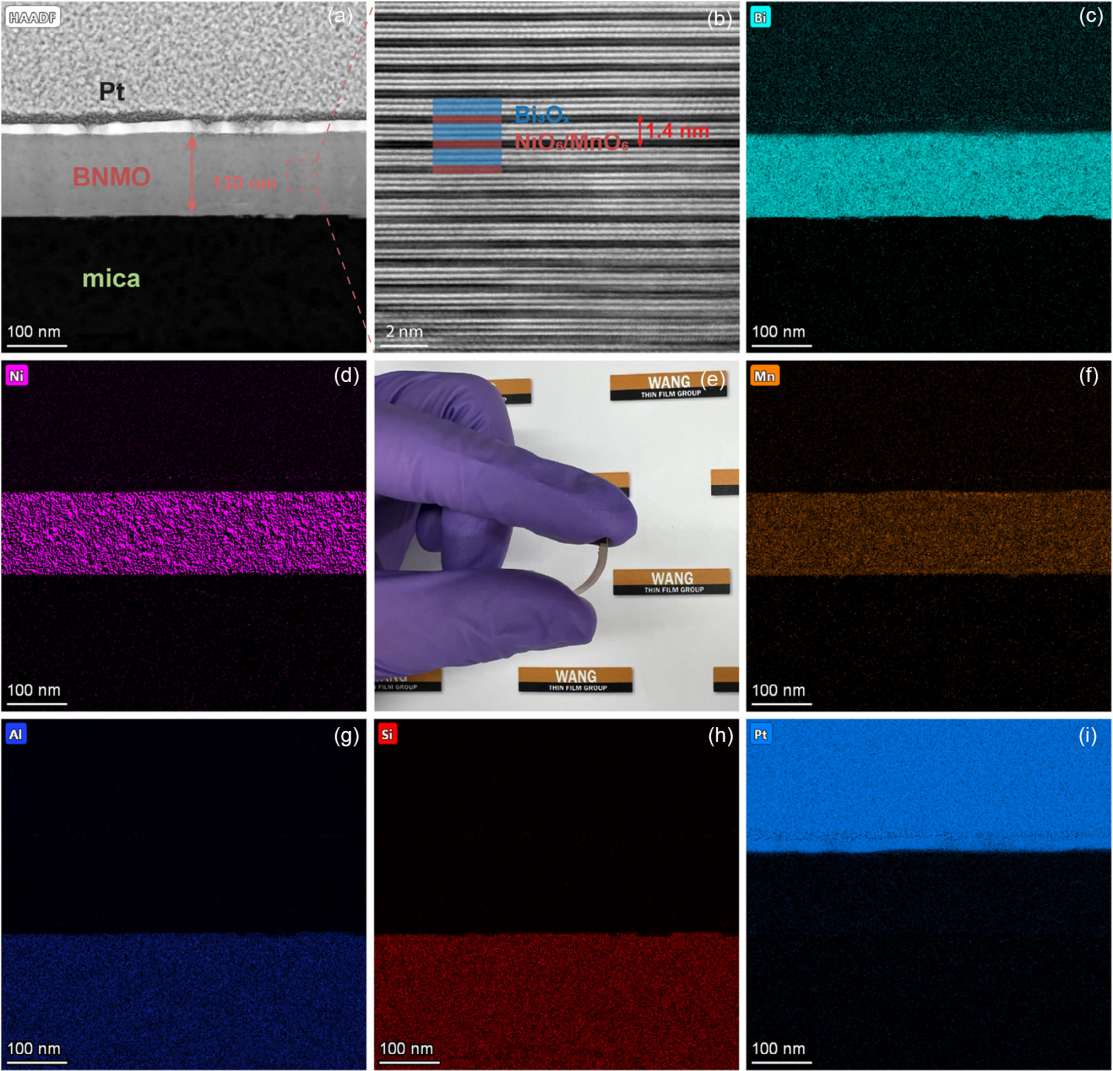
a) A low‐mag high‐angle annular dark‐field (HAADF)–STEM image of the sample prepared by FIB. The BNMO layer has a thickness of 130 nm. b) A HRSTEM image of the BNMO film that shows the LSC structure. The interlayer spacing is determined to be 1.4 nm. c,d,f–i) The EDS mapping images that correspond to the HAADF image in (a). e) A photo that shows the great flexibility of the mica sample.

The microstructure and chemical composition of BNMO film, as grown on mica, have been meticulously characterized using STEM/energy‐dispersive X‐ray (EDX), as depicted in **Figure** **2**. Figure 2a displays a low‐magnification cross‐sectional STEM image of the sample, revealing the BNMO film's thickness to be approximately 130 nm. A protective Pt layer was coated on the film to safeguard it during the sample thinning process using focused ion beam (FIB). A high‐resolution STEM (HRSTEM) image taken from the dashed square area is presented in Figure 2b. This image distinctly showcases the intrinsic LSC structure of the BNMO film, aligning with the archetypal structures documented in prior research.^[^ ^16^, ^23^ ^]^ Specifically, the supercell structure is characterized by two distinct oxide slabs: Bi_3_O_*x*_ and a combination of NiO_6_/MnO_6_. These slabs are organized in an alternate stacked arrangement in the out‐of‐plane (OP) (00 L) direction, and the measured interlayer spacing is 1.4 nm, as displayed in Figure 1b. A striking feature evident from the images is the sharp and clean interface between the mica and the film. This absence of noticeable interdiffusion underscores the superior crystalline quality of the film. Nonetheless, there's a discernible distortion at the base of the film, likely an artifact from the gallium‐focused ion beam, hinting at some localized damage during sample preparation. A deeper insight into the sample's elemental composition is provided by the energy‐dispersive X‐ray spectroscopy (EDS) mapping images, as illustrated in Figure 2c–i. The delineated interfaces between the characteristic elements of the mica and the film further substantiate our prior observations. This, in conjunction with the XRD result showcased in Figure 1b, confirms the successful epitaxial growth of the BNMO film with an LSC structure on the mica substrate. The significance of maintaining the LSC structure, which is crucial for preserving the multiferroic properties of the BNMO film, will be elaborated upon in the subsequent sections.

**Figure 3:**
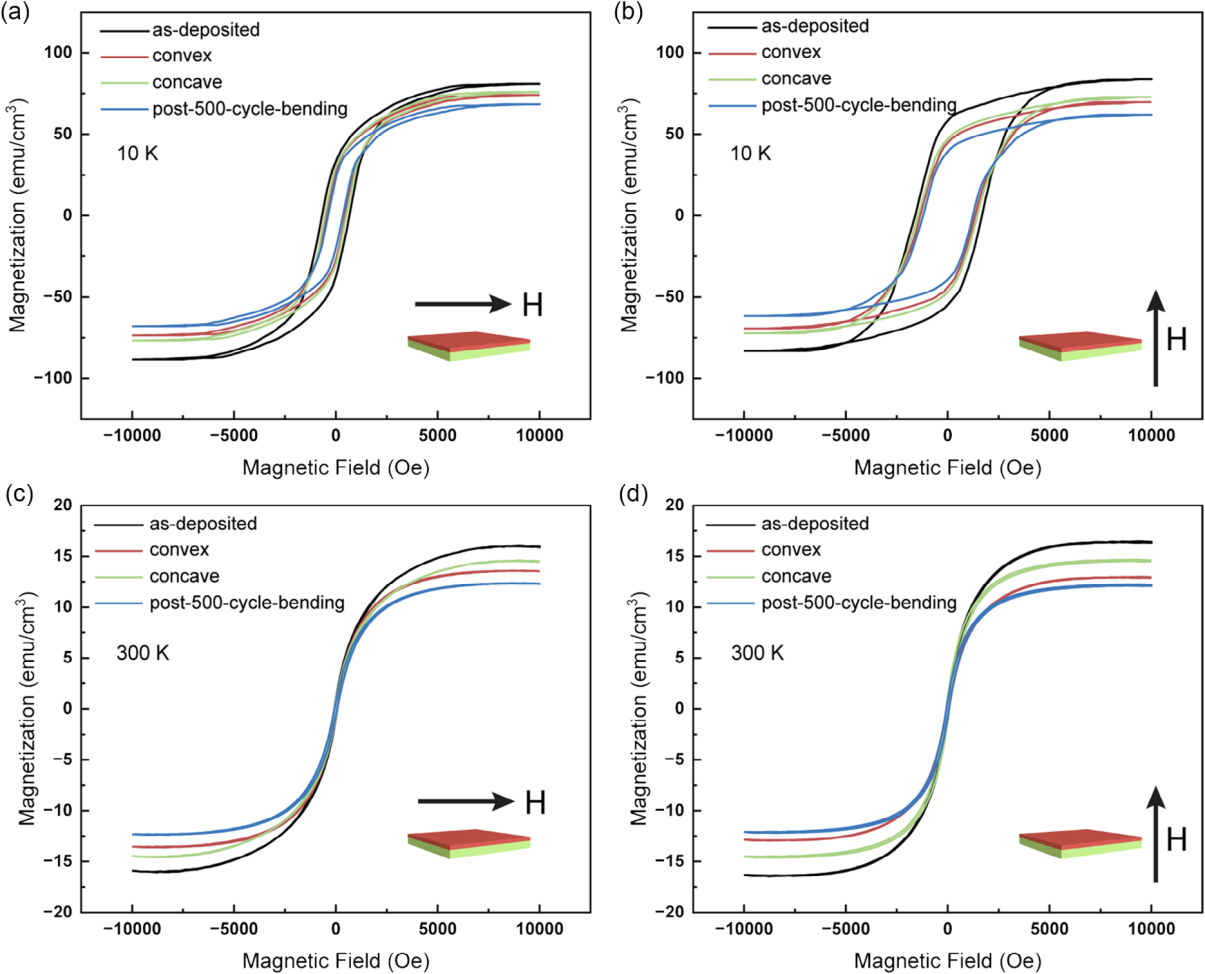
M–H hysteresis loops were measured for the as‐deposited sample, the convex sample, the concave sample, and the post‐500‐cycle‐bending sample at different conditions. The measurement conditions are a) 10 K and IP direction, b) 10 K and OP direction, c) 300 K and IP direction, and d) 300 K and OP direction, respectively.

BNMO has been documented to exhibit magnetic properties, with its magnetism primarily originating from the double‐exchange interaction among the Ni and Mn ions.^[^ ^22^, ^49^ ^]^ The magnetic properties of the BNMO film, deposited on a mica substrate under four distinct conditions, were probed using magnetization versus field (M–H) hysteresis loops. Both 300 K and 10 K temperature measurements were performed, with the 10 K data being collected post field cooling at 1 T. The M–H characterizations were executed both in the in‐plane (IP) and in the OP directions, applying a magnetic field of up to 10 000 Oe. These results are represented in **Figure** **3** and tabulated in detail in **Table** **1**. Upon comparing Figure 3a with b, a distinct difference in coercive field (*H*_c_) is noted. Specifically, the IP direction consistently exhibits a lower *H*_c_ than the OP direction at 10 K across all conditions. Consequently, the IP direction can be identified as the film's preferred anisotropy or easy axis. To numerically express this magnetic anisotropy, the difference in *H*_c_ values across the conditions was computed using the formula $\Delta H_{c}=(H_{c,\text{ OP}}-H_{c,\text{ IP}})/H_{c,\text{ OP}}$. The resultant $\Delta H_{c}$ values for the as‐deposited, convex, concave, and post‐500‐cycle‐bending samples are 66.2%, 66.4%, 66.6%, and 66.5%, respectively. While a consistent magnetic anisotropy is observed across all the conditions, there's a noticeable decline in *H*_c_ values, with the largest reduction observed after 500 bending cycles. This decreasing trend, estimated to be 11.9% and 11.2% for IP and OP respectively, might be ascribed to changes in grain orientations induced by the mechanical deformations, especially considering the mica substrate's flexibility. Interestingly, the saturation magnetization (*M*_s_) remains relatively stable in both IP and OP orientations, with any minor discrepancy likely due to calibration errors during data processing. This observation aligns with the established understanding that a magnetic material should manifest consistent saturation values when all its domains are coherently aligned. However, a decline in *M*_s_ values is evident upon subjecting the sample to mechanical deformations, suggesting potential microstructural damage incurred during the deformation process. Notably, at 300 K, the magnetic properties of the sample undergo a pronounced transformation. While the overarching trend remains—with the IP direction retaining its position as the easy axis and both *H*_c_ and *M*_s_ values declining upon curving the sample—the absolute values for *H*_c_ and *M*_s_ measured at 300 K exhibit a substantial drop in comparison to their 10 K counterparts. For instance, in the as‐deposited sample, *M*_s_ reduces from ≈87 emu cm^−3^ (averaged over IP and OP) at 10 K to 16 emu cm^−3^ at 300 K. Concurrently, *H*_c_ values drop from 470 Oe (IP) and 1392 Oe (OP) at 10 K to 62 Oe (IP) and 84 Oe (OP) at 300 K. Additionally, the $\Delta H_{c}$ values decrease to 26.2%, 24.7%, 25.6%, and 24.3% across the four conditions. Hence, the ferromagnetic property of the samples generally exhibits a reduced response at 300 K in comparison to their more pronounced manifestation at 10 K. Similar results were reported in previous studies as well.^[^ ^6^, ^8^, ^16^ ^]^ In comparison to the BNMO film on the STO substrate, our sample on mica exhibits enhanced magnetic saturation and more pronounced anisotropic behavior.^[^ ^23^ ^]^ This enhancement in magnetic properties could be possibly attributed to the distinct crystallographic orientation and strain relaxation induced by the vdW epitaxy mechanisms inherent to the film on mica substrate. The unique layered structure of the mica facilitates better alignment and orientation of the magnetic domains in the BNMO film, leading to increased magnetic saturation. Additionally, the inherent vdW epitaxy and the flexibility of the mica substrate allow for effective strain relaxation, which further contributes to the pronounced anisotropic magnetic behavior observed in our samples. Given these superior magnetic properties combined with its inherent flexibility, this sample emerges as a promising candidate for flexible spintronic device applications.

**Table 1:** Comparison of the magnetic properties of the samples under different bending conditions

	As‐deposited				Convex				Concave				Post‐500‐cycle‐bending			
	10 [K]		300 [K]		10 [K]		300 [K]		10 [K]		300 [K]		10 [K]		300 [K]	
	IP	OP	IP	OP	IP	OP	IP	OP	IP	OP	IP	OP	IP	OP	IP	OP
*M*_s_ [emu cm^−3^]	89	85	16	16	74	70	14	13	77	72	15	15	67	62	12	12
*H*_c_ [Oe]	470	1392	62	84	433	1290	55	73	446	1334	58	78	414	1236	53	70

**Figure 4:**
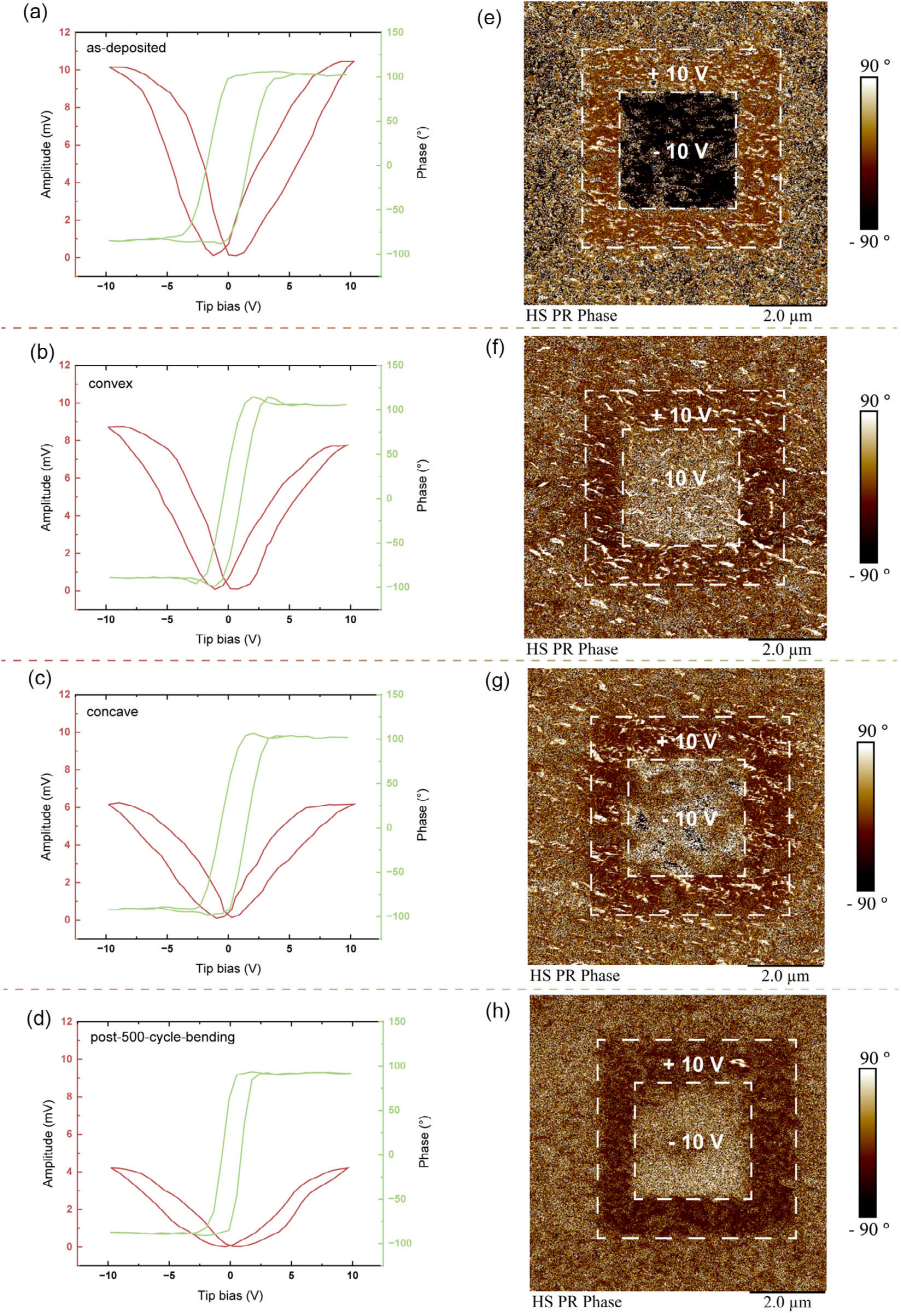
Phase and amplitude switching curves for a) the as‐deposited sample, b) the convex sample, c) the concave sample, and d) the post‐500‐cycle‐bending sample. Phase mapping image for e) the as‐deposited sample, f) the convex sample, g) the concave sample, and h) the post‐500‐cycle‐bending sample.

Considering its single‐phase room‐temperature multiferroic properties, the ferroelectric properties of BNMO on mica were investigated under all four bending conditions. It's essential to note that the source of this ferroelectricity predominantly lies in the lone pair electrons found in the 6s orbitals of the Bi ions.^[^ ^12^, ^22^ ^]^ To delve deeper into these properties, piezoresponse force microscopy (PFM) was utilized, enabling the acquisition of both phase mapping images and ferroelectric switching curves. The measurement procedure entailed an initial writing phase, wherein a 5 × 5 μm square was subject to polarization in one direction with a +10 V bias, succeeded by the polarization of a 3 × 3 μm square in the opposing direction using a −10 V bias. Following this polarization, the PFM tip was employed to read the polarization states within these domains, revealing a stark contrast in the polarized regions. The phase and amplitude switching curves were derived by subjecting the sample to a sweeping bias ranging from −10 to 10 V. These outcomes are depicted in **Figure** **4**. It is evident from the data that the sample consistently retains its ferroelectric characteristics across varied bending conditions, demonstrating resilience and adaptability. A closer inspection of the domain mapping images spanning the four conditions (Figure 4e–h) reveals that the as‐deposited sample offers the best contrast. The reduced clarity in the mapping images of the other three conditions can likely be attributed to microstructural alterations and measurement noise, the latter particularly stemming from minor sample vibrations observed in the convex and concave samples. Moreover, the amplitude switching curves (Figure 4a–d) distinctly exhibit a characteristic butterfly shape, while the phase switching curves present a hysteresis loop across all conditions. To evaluate the ferroelectric retention properties, PFM mapping was performed by acquiring the domain mapping image 3 days after poling (shown in Figure S8, Supporting Information) and the result shows comparable mapping results, indicating a reasonable retention behavior of the sample. These observations highlight the robust ferroelectric response of the samples across various bending scenarios, positioning it as a promising candidate for flexible ferroelectric device applications.

**Figure 5:**
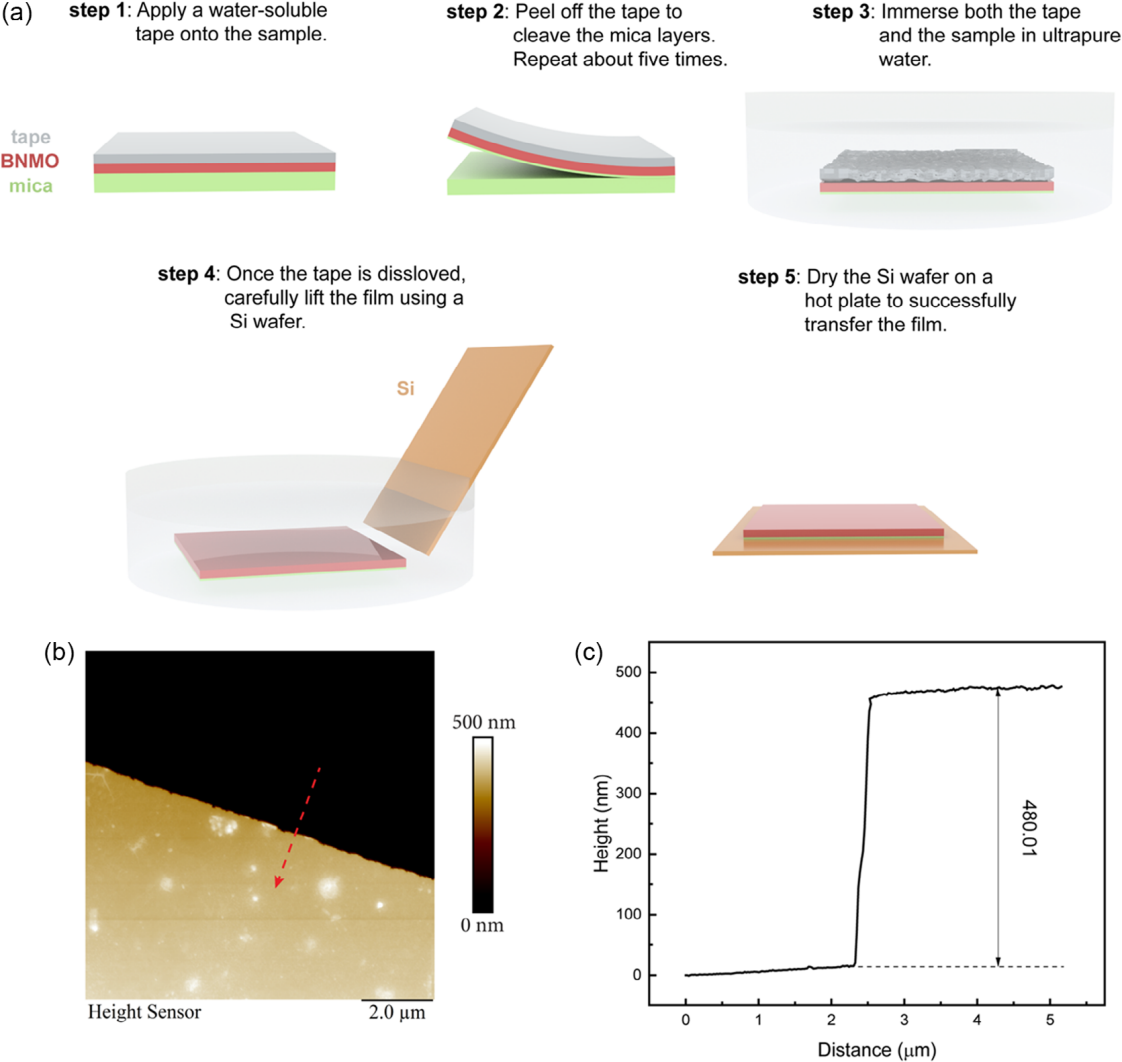
a) Schematic drawings show the transfer process, and the texts elaborate the transfer details. b) An AFM image shows the surface topography of the post‐transfer sample. c) A height profile plot based on (b) shows the thickness of the post‐transfer sample.

Previous studies have documented the ability to produce mica flakes with thicknesses spanning from several hundred nanometers down to a few nanometers.^[^ ^42^ ^]^ This is achieved by pressing a poly(dimethyl)‐siloxane (PDMS) stamp against bulk mica and then abruptly peeling it away. A similar PDMS‐based method was initially adopted to thin down the samples. However, it did not work well in this case, which could be attributed to the low adhesion between the PDMS stamp and the sample surface. In some cases, PDMS was able to peel off the mica flakes. However, the yield was very low. Alternatively, we adopted a method using a water‐soluble tape to reduce the mica thickness to a few hundred nanometers, all performed while ensuring the integrity and completeness of the BNMO film. Notably, the thinned sample can be seamlessly transferred to a different substrate (in this case, Si), without hindering subsequent property measurements. The thinning and transfer process is illustrated in **Figure** **5**a. Initially, a water‐soluble tape is affixed to the film side, followed by uniform pressing. Upon peeling off the tape, the film remains adhered to it while a section of the mica gets cleaved. This observation suggests that the interlayer bonding forces within the mica are weaker than both the bond between the film and mica and the bond between the film and tape. To ensure continuous thinning of the mica substrate, this procedure is repeated five times. Subsequently, the tape, along with the adhered sample, is immersed in ultrapure water. As the tape swiftly dissolves, the remaining BNMO film and residual mica are left floating. Using a Si wafer, the sample is carefully lifted, and the wafer is then dried atop a hot plate. This process successfully transfers the sample onto the Si wafer. The thickness of the relocated sample is ascertained using atomic force microscopy (AFM), revealing a commendably smooth surface, as depicted in the tomographic image (Figure 5b). Drawing a line across both the mica and the film in Figure 5b and subsequently plotting the height profile yield the graph in Figure 5c. Upon analysis, the total thickness of the film/mica stack is determined to be 480 nm. Within this, the film contributes 130 nm while the mica accounts for 350 nm, as shown in Figure S3, Supporting Information. In our study, as a demonstration, we repeated step 2 five times, resulting in a residual mica layer with a thickness of 350 nm. Intriguingly, by varying the number of repetitions of step 2, the thickness of the residual mica layer can be adjusted, as demonstrated in Figure S4, Supporting Information. A precise control over its thickness remains a challenge for now because the number of mica layers that can be peeled off every time depends on many factors such as the applied force, the uniformity of the contacting interface, and the remaining sample thickness, etc., and continuous efforts will be spent on improving the thinning process to achieve a more controlled and precise technique. Nonetheless, this offers a straightforward, uncontaminated, and adaptable approach suited for a range of application contexts. The advantage of the technique lies in its ability to reliably achieve the desired thickness of the mica layers without compromising the structural and functional integrity of the BNMO film, and the yield is also higher than that of the PDMS‐based approach. After the transfer, the sample surface and the microstructure were meticulously analyzed using optical microscopy and HRSTEM, with the results depicted in Figure S5, Supporting Information. It is worth emphasizing that the film shows intact post‐transfer. Moreover, its layered structure closely mirrors that of its pretransfer counterpart, underscoring the efficacy of our transfer technique and the robust nature of the BNMO films. To verify the film properties post‐transfer, we conducted a comparative analysis of the magnetic properties between the as‐deposited and post‐transfer samples. As illustrated by Figure S6, Supporting Information, the transferred film exhibits a pronounced ferromagnetic hysteresis loop, albeit with a minor reduction in the saturation magnetization. A comprehensive data comparison is available in Table S1, Supporting Information.

The successful integration on mica and post‐transfer to Si of BNMO LSC films presents several important findings. First, regarding the vdW epitaxy growth of LSC oxides, it is interesting to note that BNMO grows on mica very well with nearly perfect layered structures and abruptly sharp interfaces as evidenced by the XRD and STEM analysis. Compared to previously reported oxide integrations, such as VO_2_, BaTiO_3_‐Fe, and BaZrO_3_‐Co,^[^ ^43^, ^46^, ^50^ ^]^ the vdW epitaxial growth of LSC presents surprisingly high quality. It has been previously reported that high substrate strain is required for the growth of LSC structures, and thus LAO substrates or a typical buffer layer stack on STO is required for the successful growth of LSC layers.^[^ ^47^ ^]^ It is intriguing to observe the successful growth of epitaxial LSC directly on mica under the loose vdW bonding without such strong interfacial strain required. It is possible that the layered nature of mica and stacking sequence favors the layered BNMO growth.^[^ ^51^ ^]^ Secondly, the robustness of BNMO films and their properties is of technological importance. Undergoing the aggressive bending condition on mica, the film structure and physical properties remain intact. Even after the subsequent mica thinning process, the films still remain intact. This is essential for the practical transfer of these LSC films onto device substrates. Compared to other prior oxide transfer work via a water‐soluble sacrificial layer Sr_3_Al_2_O_6_, the transfer method shown here by peeling film directly off mica substrate is more straightforward and provides a more robust performance in terms of the film properties.^[^ ^23^, ^38^ ^]^ Finally, the demonstration of the unique mica thinning process with thickness control paves an avenue for LSC film transfer on other substrates with reliable film growth quality. These are all important demonstrations toward future practical applications of multiferroic BNMO and others LSC films on advanced flexible electronics using either mica or other flexible substrates as the device platform.

## 3 Conclusions

The potential of BNMO LSC films deposited on mica substrates for flexible device applications was rigorously examined, providing pivotal insights into their properties under distinct mechanical conditions. The inherent weak vdW bonding in the interlayers of mica allows for the processing of ultrathin and flexible mica layers. This vdW feature also enables the epitaxy of multiferroic BNMO films, regardless of the required stringent strain condition, and presents unique opportunities for developing next‐generation flexible devices. Detailed STEM/EDX analyses reveal unambiguous layered structure and the sharp boundary with mica underscores the high quality and effectiveness of the vdW epitaxy process. Such sharp interface is indicative of a potential reduction in interface‐related defects, which are key to film properties. The magnetic and ferroelectric characterizations of the films demonstrate the persistent magnetic properties and a robust ferroelectric response, even post mechanical deformation. These demonstrations open up avenues for the utilization of BNMO in flexible devices. Furthermore, the innovative technique developed for thinning the mica substrate and transferring the BNMO film on Si and other substrates is of great technological importance. Finally, while the scope of this study primarily focuses on the fundamental characterization of the magnetic and ferroelectric properties of the BNMO films under various mechanical conditions, the integration of these materials into actual device applications is highly desirable as suggested by previous studies.^[^ ^52^ ^]^ As a result, future efforts should be invested in this avenue, bridging the gap between fundamental research and practical device implementation and enabling the potential of the flexible materials in the realm of spintronics and flexible electronics.

## 4 Experimental Section

4.1

4.1.1

##### Thin‐Film Growth

The BNMO target was prepared through a solid‐state mixing method. Stoichiometric amounts of Bi_2_O_3_, NiO, and Mn_2_O_3_ powders were combined, pressed into a one‐inch pellet, and then sintered in a furnace at 750 °C for 3 h in an air atmosphere. The BNMO films were grown on muscovite mica substrates measuring 2 × 2 cm using the pulsed laser deposition technique with a KrF excimer laser (Lambda‐Physik COMPex Pro 205, *λ* = 248 nm). The film growth occurred at 700 °C under an oxygen pressure of 200 mTorr, utilizing a laser fluence of 3.6 J cm^−2^ and a laser frequency of 2 Hz. After deposition, the films underwent annealing at 400 ˚C with an oxygen pressure of 500 Torr for 1 h before cooling down to room temperature at a rate of 10 °C min^−1^. This annealing process was crucial for achieving optimal crystalline quality and oxygen stoichiometry of the film. Annealing in an oxygen‐rich environment aids in recovering oxygen loss during deposition and ensuring a stoichiometric balance of the film. The chosen cooling rate of 10 °C min^−1^ was instrumental in preventing the formation of cracks and internal stresses within the film, which can occur due to thermal shock in rapid cooling processes.

##### Microstructure Characterization

The microstructure and crystallinity of the samples were analyzed using various techniques. XRD was performed on a PANalytical Empyrean instrument, while TEM and high‐resolution STEM were conducted on a Thermo Fisher Scientific TALOS 200X operated at 200 kV. For the TEM samples, fabrication was done using a Quanta 3D FEG dual‐beam scanning electron microscope with a gallium FIB. To protect the sample from ion beam damage, a thin Pt protection layer was deposited on the top surface. Chemical composition analysis was carried out using EDS mode in the STEM. Additionally, optical images were captured using an optical microscope equipped with AmScope software.

##### Physical Properties Measurements

The ferroelectric properties of the samples were characterized using a Bruker Dimension Icon AFM equipped with a conductive Pt‐/Ir‐coated Si tip (SCM‐PIT V2). To investigate the magnetic properties of the samples, a vibrating sample magnetometer mode of the Magnetic Property Measurement System (MPMS3 by Quantum Design) was utilized. The magnetization versus magnetic field (M–H) curves were acquired both IP and OP directions. Before obtaining accurate results, potential calibration errors were addressed. High‐field slope and magnetic remanence were corrected using a built‐in script provided by the instrument. Additionally, the sample geometry factor was considered to ensure precise measurements. The multiplicative factor for the sample geometry was determined to be in the range of 0.9–1.2 using a sample geometry calculator. The optical properties of the samples were measured using a UV–vis–NIR Lambda 1050 spectrometer. Data on transmittance were collected and subsequently transformed into absorption values through the relation: $\alpha =\ln\left(\frac{1}{T}\right)t$, where *α* denotes the absorption coefficient, *T* is the measured transmittance, and *t* represents the film thickness. The optical bandgap was determined using Tauc's relation, expressed as (*αhν*) ∝ ${\left(h\nu \right)}^{n}$, where *n* equals $\frac{1}{2}$ for direct bandgap.

##### Film Transfer Process

A water‐soluble tape obtained from SmartSolve (Bowling Green, OH 43 402) was employed to facilitate the peeling off the thin film from the mica substrates. The water‐soluble tape used biodegradable materials and low‐tack water‐soluble adhesive to ensure complete water solubility, and it was a paper‐based material made of wood pulp and other compostable materials that ensured complete dissolution in 30 s or less. In the practice of dissolution, the sample was soaked in room‐temperature ultrapure water for 1 min to ensure complete dissolution. The transfer process involving this tape was thoroughly described in the main texts, providing a comprehensive account of the methodology used for detaching the thin film from the mica substrates.

## Conflict of Interest

The authors declare no conflict of interest.

## Author Contributions

The idea for this research project was conceived by J.S., B.T., and H.W. They collectively designed all the experiments and developed the mechanistic understanding behind the study. J.S. and B.T. made equal contributions to the project. J.S. took charge of the X‐ray diffraction characterization work and was responsible for creating the conceptual drawings. The scanning transmission electron microscopy and energy‐dispersive spectroscopy characterization work was conducted by J.S., K.X., and Y.Z. working together. The transfer process of the films was carried out jointly by J.S. and B.T., J.S. conducted the magnetic measurements and performed the piezoresponse force microscopy characterization work. The experimental funding for the project was acquired by H.W. The manuscript was mainly written by J.S. with contributions and assistance from the other co‐authors. All authors have reviewed and approved the final version of the manuscript.

## Supporting information

Supplementary Material

## Data Availability

The data that support the findings of this study are available in the supplementary material of this article.
