# Supplementary material for: Van der Waals Epitaxy of Bismuth‐Based Multiferroic Layered Supercell Oxide Thin Films Integrated on Flexible Mica Substrate
Source: Small Sci. 2023 Dec 28;4(2):2300244. doi: 10.1002/smsc.202300244 (PMC11935282; doi:10.1002/smsc.202300244)
Supplement: Supplementary file 1 — Supplementary Material [file SMSC-4-2300244-s001.pdf]

Supplementary Materials

Van der Waals Epitaxy of Bismuth-based Multiferroic Layered Supercell Oxide Thin Films Integrated on Flexible Mica Substrate

Jianan Shen, [a] *Benson Kunhung Tsai,* [a] *Yizhi Zhang,* [a] *Ke Xu,* [a] *James P. Barnard,* [a] *Zedong Hu,* [b] *Xinghang Zhang,* [a] *Haiyan Wang*[*ab]

a School of Materials Engineering, Purdue University, West Lafayette, Indiana 47907, United States.

b Elmore Family School of Electrical and Computer Engineering, Purdue University, West Lafayette, Indiana 47907, United States.

*Corresponding author email address: hwang00@purdue.edu

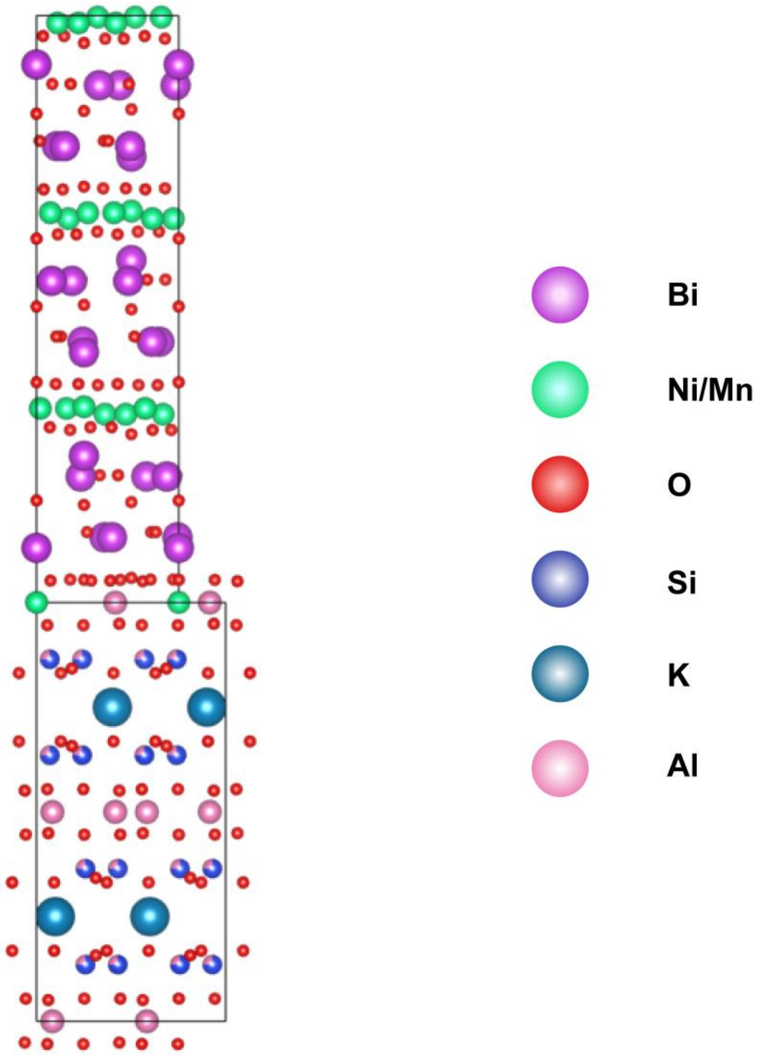

Figure S1. A crystal model of the BNMO LSC film grown on the mica substrate.

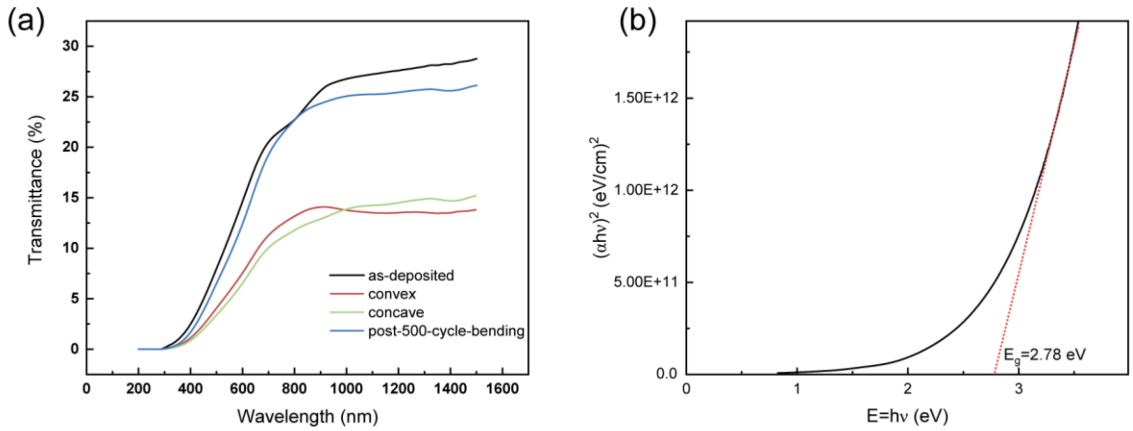

Figure S2. (a) Transmittance spectra of the samples under four different bending conditions. (b) Tauc's plot extracted from (a) for the determination of the bandgap.

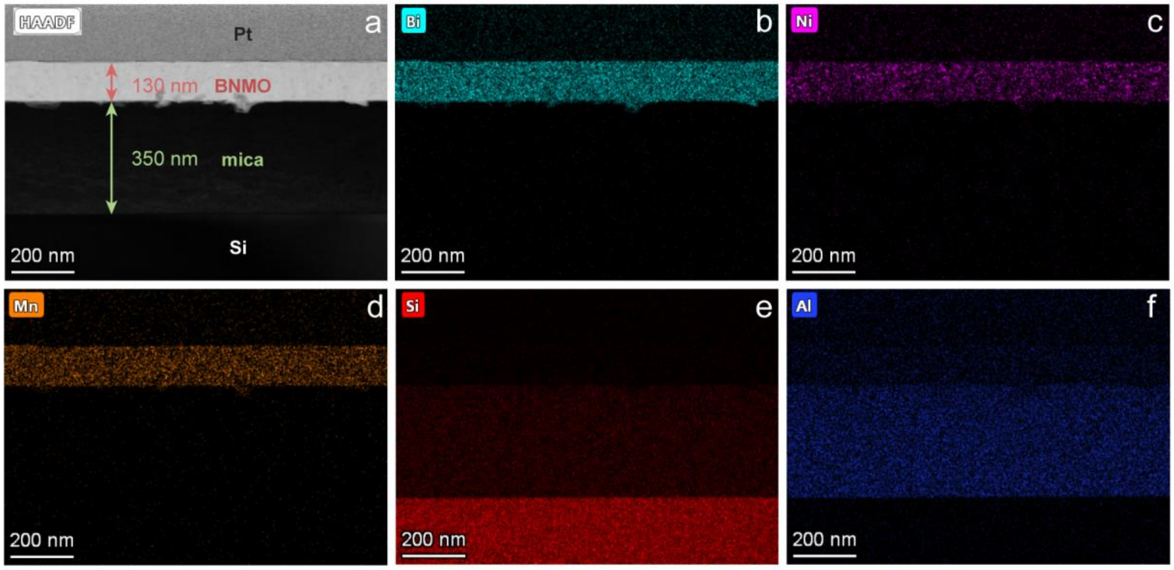

Figure S3. (a) HAADF-STEM image showcasing the post-transfer film/mica sample on a Si wafer. (b-f) EDS maps corresponding to the region depicted in (a).

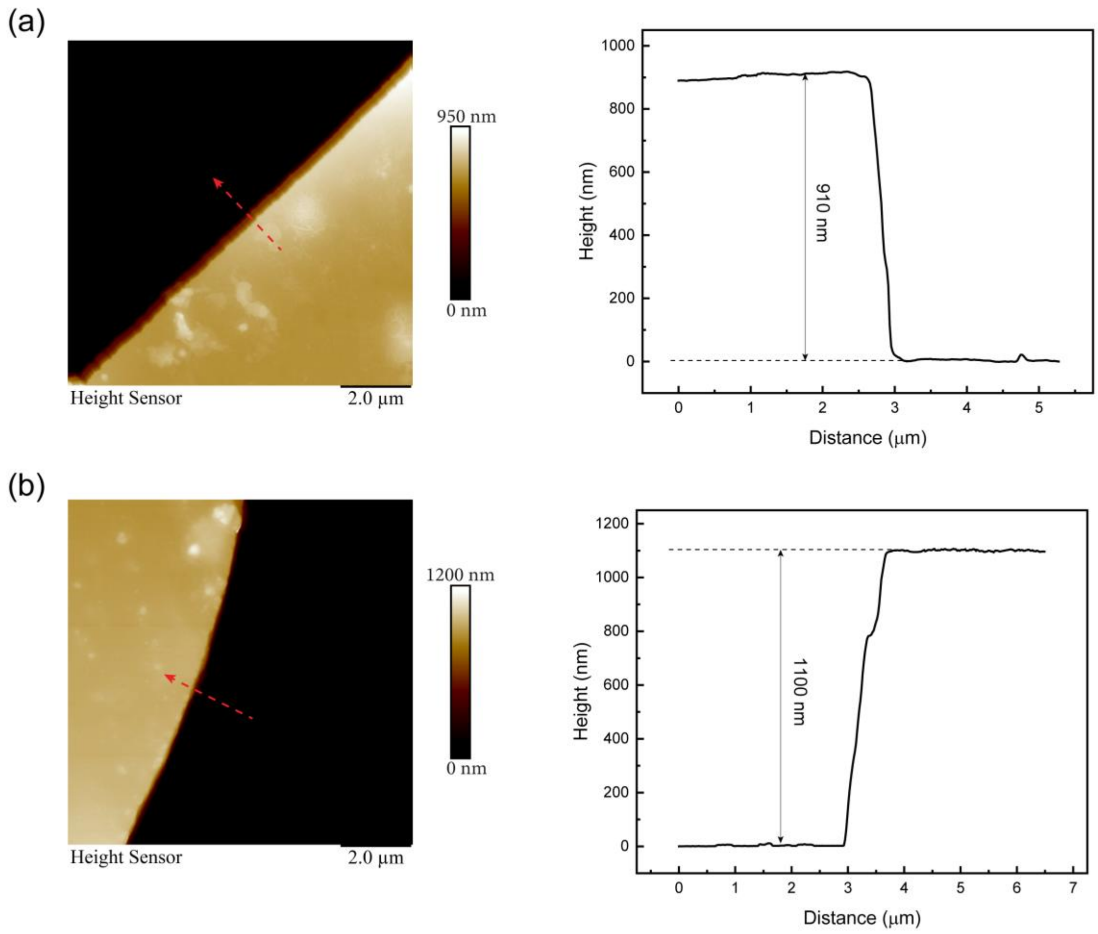

Figure S4. (a) AFM topographical image of a post-transfer sample, indicating a thickness of 910 nm from the height profile. (b) AFM topographical depiction of a thicker post-transfer sample, with the height profile revealing a thickness of 1100 nm.

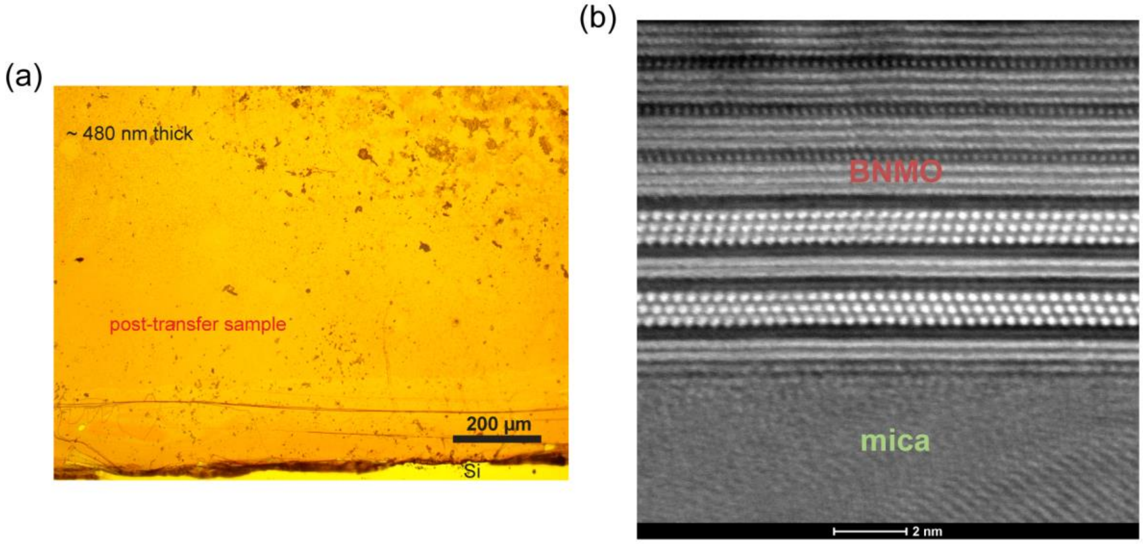

Figure S5. (a) Optical microscopy image of the post-transfer sample, showcasing a thickness of 480 nm. (b) HRSTEM image of the post-transfer sample, highlighting the intact LSC structure.

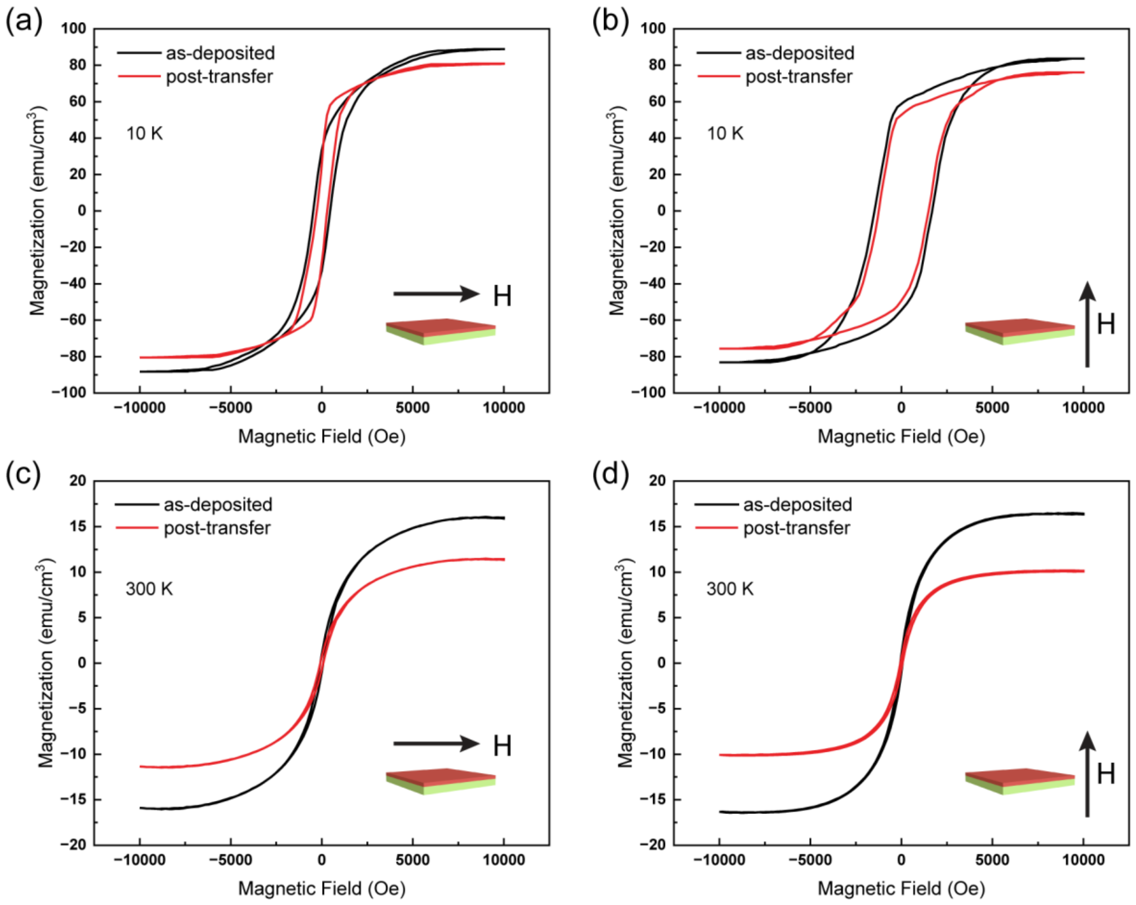

Figure S6. (a-b) M-H hysteresis loops captured at 10 K for the as-deposited and post-transfer samples in the IP and OP orientations, respectively. (c-d) M-H hysteresis loops taken at 300 K for the as-deposited and post-transfer samples in the IP and OP orientations, respectively.

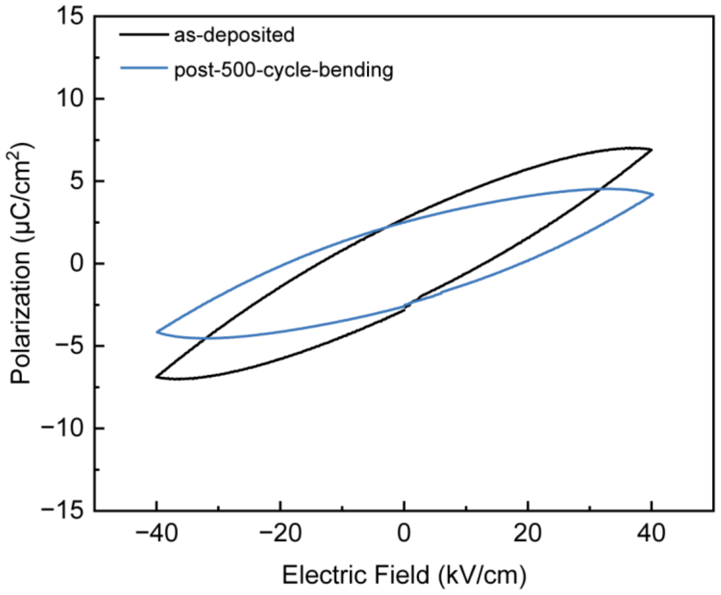

Figure S7. Polarization versus electric field plot of the as-deposited sample and post-500-cycle-bending sample, respectively.

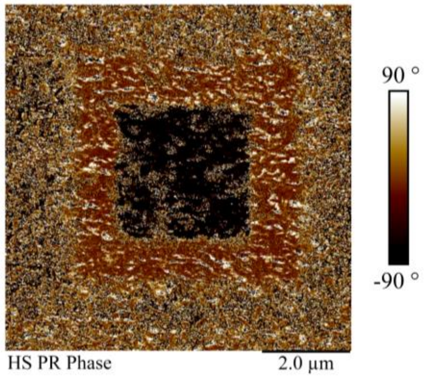

Figure S8. The PFM domain mapping image of the as-deposited sample acquired three days after poling.

Table S1. Comparison of the magnetic properties of the as-deposited sample and post-transfer sample.

	As-deposited				Post-transfer			
	10 K		300 K		10 K		300 K	
	IP	OP	IP	OP	IP	OP	IP	OP
M_s (emu/cm^3)	89	85	16	16	80	76	11	10
H_c (Oe)	470	1392	62	84	452	1350	60	82
